# The neural transfer effect of working memory training to enhance hedonic processing in individuals with social anhedonia

**DOI:** 10.1038/srep35481

**Published:** 2016-10-18

**Authors:** Xu Li, Zhi Li, Ke Li, Ya-wei Zeng, Hai-song Shi, Wen-lan Xie, Zhuo-ya Yang, Simon S. Y. Lui, Eric F. C. Cheung, Ada W. S. Leung, Raymond C. K. Chan

**Affiliations:** 1 Neuropsychology and Applied Cognitive Neuroscience Laboratory, CAS Key Laboratory of Mental Health, Institute of Psychology, Beijing, 100101, China; 2 University of Chinese Academy of Sciences, Beijing, 100048, China; 3 Key Laboratory of Adolescent Cyberpsychology and Behavior(CCNU), Ministry of Education, School of Psychology, Central China Normal University, Wuhan, 430079, China; 4 MRI Center, Hospital 306, 100101, Beijing, China; 5 North China Electric Power University, Beijing, 102206, China; 6 Castle Peak Hospital, Hong Kong Special Administration Region, China; 7 Department of Occupational Therapy & Institute of Neuroscience and Mental Health, University of Alberta, T6G 2G4, Canada

## Abstract

Anhedonia, the diminished ability to experience pleasure, is a challenging negative symptom in patients with schizophrenia and can be observed in at-risk individuals with schizotypy. Deficits in hedonic processing have been postulated to be related to decreased motivation to engage in potentially rewarding events. It remains unclear whether non-pharmacological interventions, such as cognitive training, could improve anhedonia. The present study aimed to examine the neural mechanism for alleviating hedonic deficits with working memory (WM) training in individuals with social anhedonia. Fifteen individuals with social anhedonia were recruited and received 20 sessions of training on a dual *n*-back task, five sessions a week. Functional imaging paradigms of the Monetary Incentive Delay (MID) and the Affective Incentive Delay (AID) tasks were administered both before and after the training to evaluate the neural transfer effects on hedonic processing ability. Enhanced brain activations related to anticipation were observed at the anterior cingulate cortex, the left dorsal striatum and the left precuneus with the AID task, and at the dorsolateral prefrontal cortex and the supramarginal gyrus with the MID task. The present findings support that WM training may improve monetary-based and affective-based hedonic processing in individuals with social anhedonia.

Anhedonia refers to the diminished ability to experience pleasure and constitutes one of the core features in negative symptoms of schizophrenia^1^ and major depressive disorder^2^. The recently proposed Research Domain Criteria (RDoC) also includes hedonic capacity as a trans-diagnostic domain between schizophrenia and depression^3^. In patients with schizophrenia, the severity of anhedonia contributes to decreased social functioning^4^ and diminished quality of life^5^. Anhedonia is also present in non-psychotic first-degree relatives of patients with schizophrenia^6^ and individuals with psychometrically-defined schizotypy^7^, and has been considered a possible vulnerability marker of schizophrenia^8^.

Dissociation between anticipatory and consummatory pleasure experience has been demonstrated, and specific deficits in anticipatory pleasure experience have been reported in people with schizophrenia and schizotypy^9^. This anticipatory pleasure deficit has been associated with difficulties in the formation, maintenance and retrieval of affective and value representations^10^. Patients with schizophrenia showed lower self-report emotional intensity^11^ and reduced brain activations at the prefrontal cortex, the anterior cingulate cortex, the putamen and the thalamus^12^ during the maintenance of emotional representation. Working memory (WM) is responsible for information maintenance and manipulation in a short period, and thus it has been suggested as a possible underlying cognitive mechanism for anticipatory pleasure and goal-related behaviours^13^. Baddeley *et al*.^14^ have suggested the existence of a hedonic detector system within the WM model. The overlap in recruitment of the prefrontal-striatal system in hedonic processing^15^ and WM^16^ provides further evidence to support the association between hedonic function and WM.

In the healthy population, anhedonia has also been observed in individuals with schizotypy, who are medication-free but exhibit cognitive deficits and psychotic-like experiences to some extent^17^. Psychometrically-defined schizotypy showed comparable levels of social anhedonia as patients with schizophrenia, while their levels of physical anhedonia lay intermediate between patients and healthy controls^7^. There is also evidence to suggest that elevated levels of social anhedonia may predispose an individual to the development of schizophrenia-spectrum disorders^18^. Given the above, social anhedonia may be a potential target for early detection and intervention in high-risk individuals.

Cognitive remediation therapies have been developed for patients with schizophrenia to improve cognitive function with a view to alleviate negative symptoms^19^. A recent meta-analysis suggests that patients with schizophrenia could benefit from WM training and brain regions showing activation changes showed significant overlap with healthy volunteers^20^. The primary brain regions showing benefits from WM training include the dorsolateral prefrontal cortex and the striatum. It is interesting that the prefrontal-striatal system is also important in reward processing and dysfunction of this brain network may underlie impairment in motivated behaviours in patients with schizophrenia^15^. However, few studies have specifically examined the possible beneficial effect of WM training on anhedonia. A behavioural study have explored the effects of WM training on enhancing hedonic processing abilities in individuals with social anhedonia, which found that motivated actions towards positive affective rewards were dramatically enhanced with WM training^21^. Moreover, the recruitment of individuals with schizotypy could overcome some of the confounding factors when studying patients with schizophrenia, such as the effects of antipsychotic medications, symptom severity and duration of illness.

The present study examined the potential transfer effect of WM training in alleviating hedonic deficits in individuals with social anhedonia. Domain-specific hedonic processing deficits towards monetary and affective incentives have been observed in high-risk samples with anhedonia^22^,^23^. Thus functional imaging paradigms of the Monetary Incentive Delay (MID)^24^ and the Affective Incentive Delay (AID)^22^ tasks were administered both before and after WM training to evaluate the neural transfer effects on hedonic processing ability. We hypothesized that activities in the neural correlates of hedonic processing would be altered with WM training, and dissociable training effects would be observed in the processing of affective and monetary rewards. Moreover, a control group of individuals with extremely low levels of social anhedonia was also included at baseline assessment to investigate whether brain dysfunctions in the anhedonia group could be normalized to that of controls with WM training. Finally, given the involvement of maintenance function in anticipatory pleasure experience^13^, training gains and activation changes during anticipation would be correlated to examine whether the neural activation changes related to WM training were associated with improvement in WM performance.

## Results

### Training gains

**Figure 1:**
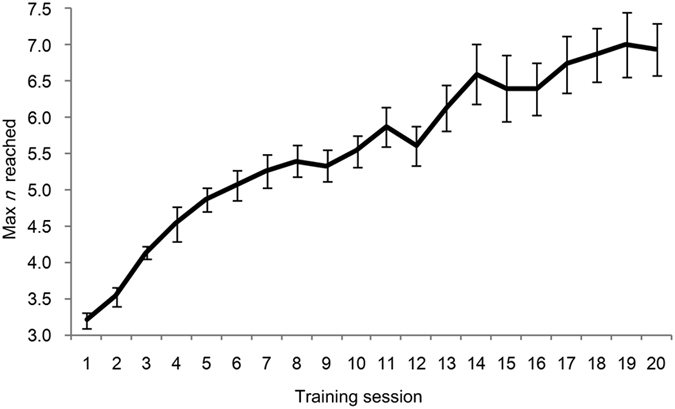
The max *n* reached on the dual *n*-back task across the 20 training sessions in the training group. Error bars represent standard error.

As shown in Fig. 1, performance in the dual *n*-back task improved continuously with training. The maximum *n*-back level reached during the first training session was 3.20 (*SD* = 0.41), which increased to 6.93 (*SD* = 1.39) in the last session. The training effect was significant (*F*(19, 266) = 24.874, *p* < 0.001). All the participants achieved a larger *n* in session 20, and two participants reached 10-back during the training while four reached 9-back. There were substantial individual differences in training benefits.

### Training benefits in other behavioural measures

The improvement in performance in the Letter-Number-Span (LNS) task was significant (*t* (14) = 3.662, *p* = 0.003); thus a general benefit on WM capacity was observed in individuals with social anhedonia. The effect on self-reported measures was also investigated with paired *t*-tests (two tailed). The reduction in Chapman Social Anhedonia Scale (CSAS) scores was significant (*t* (14) = 2.714, *p* = 0.017), with participants reporting less anhedonia after WM training, while self-reported pleasurable feelings on the Temporal Experience of Pleasure Scale (TEPS) did not change significantly after WM training (Supplementary Table S1).

### Hedonic processing deficits in individuals with anhedonia

First, behavioural performance (reaction times, RTs) in the AID and MID tasks during scanning were analyzed with 2 (Group: social anhedonia vs. controls) × 2 (Task: AID vs. MID task) × 3 (Valence: reward, punishment vs. neutral cue) repeated measures ANOVA, and the analysis revealed a significant main effect of Valence (*F*(2, 128) = 28.403, p < 0.001), with RTs in the reward (*Mean* = 226,24 ms, *SD* = 26.41) and punishment condition (*Mean* = 230.59 ms, *SD* = 25.92) shorter than in the neutral condition (*Mean* = 240.59 ms, *SD* = 22.11). No other significant main effects or interactions were found.

Brain regions showing activation differences between individuals with social anhedonia and controls in the anticipatory and consummatory phases are listed in Supplementary Table S2. Significant hyperactivation during the anticipatory and consummatory phases in the AID task were observed in the temporal and parietal cortices, including the bilateral middle temporal gyrus (MTG), the right precuneus, the superior temporal gyrus (STG), the right inferior parietal lobule (IPL), the middle frontal gyrus (MFG) and the insula. For the MID task, hypoactivations were observed in the bilateral parahippocampus, the bilateral thalamus and the right substantia nigra in the anticipatory phase in individuals with social anhedonia. Hyperactivations were found in the IPL in the anticipatory phase, and in several frontal, parietal and temporal regions as well as the right thalamus in the consummatory phase in individuals with social anhedonia.

### WM training effects on enhancing hedonic processing

**Figure 2:**
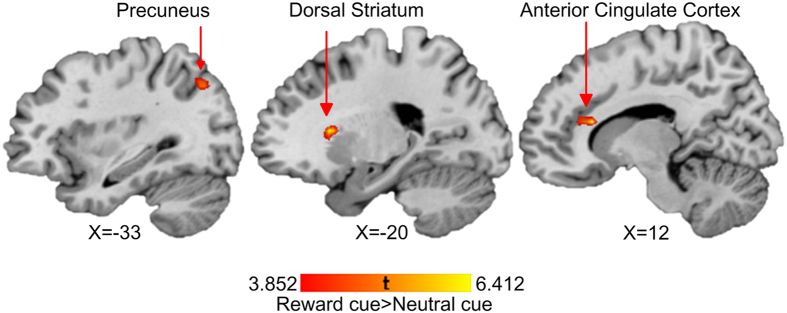
Neural training effects on the contrast of “reward cue > neutral cue” of the AID task in the anhedonia group. The brain activations in the highlighted regions were increased with WM training.

**Figure 3:**
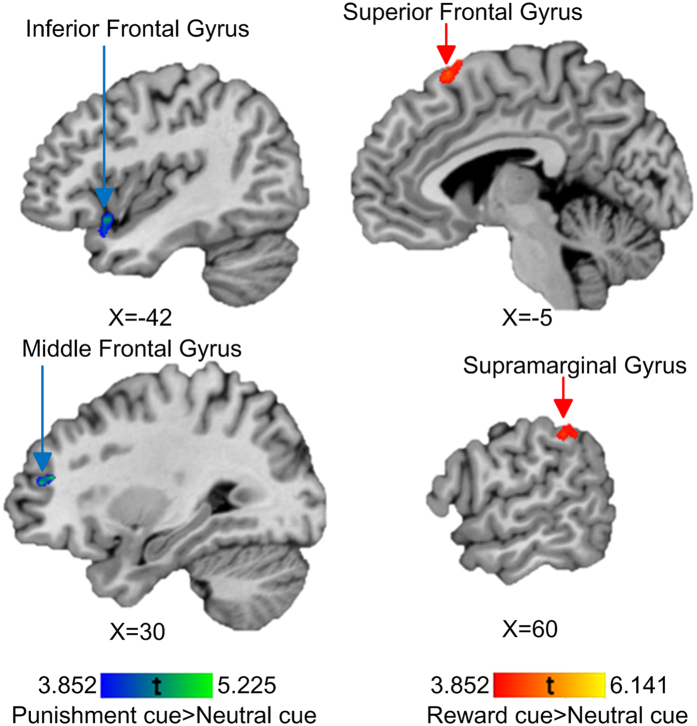
Neural training effects in the anticipation phase on the MID task in the anhedonia group, red bar represents results of the contrast of “reward cue > neutral cue”, while blue bar represents results of the contrast of “punishment cue > neutral cue”. The brain activations in the highlighted regions were increased with WM training.

Brain activation changes in the anticipatory phase of the AID and MID tasks related to WM training are listed in Table 1 and displayed in Figs 2 and 3.

In the anticipatory phase, brain activation was increased at post-training in the right anterior cingulate cortex (ACC), the left dorsal striatum and the left precuneus in the “reward cue > neutral cue” contrast of the AID task, and in the bilateral superior frontal gyrus (SFG), the right supramarginal gyrus in the “reward cue > neutral cue” contrast of the MID task. While the contrast “punishment cue > neutral cue” of the MID task was associated with increased brain activation at the right MFG and the left inferior frontal gyrus (IFG), no activation changes were found in this contrast in the anticipatory phase of the AID task.

However, activations were reduced during the consummation of both affective and monetary incentives in several frontal and parietal regions, and in some subcortical regions, including the cingulate cortex, the insula, the caudate and the bilateral parahippocampus (see Supplementary Table S3).

*Post-hoc* ROI analyses examined whether any of these changes normalized social anhedonia activation abnormalities listed in Supplementary Table S2. The majority of these activation abnormalities observed in the AID and the MID tasks in individuals with social anhedonia were not significantly different from controls following WM training, except for one cluster (x = 42, y = −60, z = 24, cluster size = 2, MTG) in the ”reward cue > neutral cue” contrast of the AID task.

### Relationship between brain function changes and behavioural improvements

The signals from the significant clusters in the anticipatory phase were extracted from the AID and the MID tasks and the correlations between signal changes in the contrasts “reward cue > neutral cue” and “punishment cue > neutral cue” and behavioural improvements on the training task were calculated.

Increase in brain activation at the SFG in the MID task was positively correlated with improvement of maximum *n* (*r* = 0.549, *p* = 0.042). Hence, a greater enhancement of WM capacity was associated with increase in activation at the SFG. No other significant correlations were found.

## Discussion

In the present study, we investigated the neural mechanism of WM training on enhancing hedonic processing abilities in individuals with social anhedonia, especially the neuroplastic effect on brain activities in the anticipation of potential gain or loss. Altered activations were observed with WM training during the anticipation of affective and monetary incentives, and dissociable training effects were found regarding the types of incentives. Specifically, increased brain activations were observed during the anticipatory phase at the ACC, the dorsal striatum and the precuneus for the AID task, while increase in activation was observed at the dorsolateral prefrontal cortex and parietal regions for the MID task. Brain activities were reduced in the consummation of both affective and monetary incentives. The observed group difference between individuals with social anhedonia and controls at baseline were no longer significant after WM training, indicating a normalization of brain activations in individuals with social anhedonia with WM training. Correlation analysis confirmed that activation increase in the SFG was positively correlated with behavioural improvement on the *n*-back task.

Individuals with social anhedonia showed hedonic processing deficit in the anticipation of positive affective contents but not monetary incentives at the behavioural level compared with controls^22^. Differences in brain activation in processing affective and monetary rewards were also observed in the present study. Hyperactivations were observed in the MTG and the precuneus in the anticipation of positive affective images, and in the STG and the insula in the consummation of positive affective contents. These regions are involved in the processing of social information, such as others’ mental states and feelings, and thus are collectively referred to as the “social brain”^25^. Positive correlations were observed between anhedonia score and activation at the MTG and the precuneus when performing social cognition tasks in individuals with social anhedonia^26^. Hyperactivations at the STG, the MTG and the precuneus were also observed during social cognition tasks in patients with schizophrenia^27^. When performing the MID task, reduced brain activations were observed at the parahippocampus, the thalamus and the substantia nigra in the anticipation of monetary rewards in the social anhedonia group. Both the parahippocampus^28^ and the thalamus^29^ are key regions related to reward processing. The substantia nigra supplies the striatum with dopamine, which may be the neurobiological mechanism of hedonic processing of reward^30^. Functional abnormalities in these brain regions in hedonic processing of monetary rewards are consistent with findings in patients with schizophrenia^31^. Moreover, studies in patients with major depressive disorder^32^, who are also characterized by prominent anhedonia, also revealed attenuated brain activations in these regions. Although the behavioural performances in the AID and the MID tasks during scanning did not differ between individuals with social anhedonia and controls, dissociable neural processing for affective and monetary information was observed. Thus investigation of the effects of different types of incentives used in reward processing is needed. The abnormal functions of these brain regions may represent a compensatory mechanism in individuals with social anhedonia to better process affective and monetary rewards.

Training related hyperactivation was found in the dorsal striatum, the ACC, and the precuneus during the anticipation of positive affective rewards. The dorsal striatum is involved in the anticipation of social and monetary rewards^33^ and is engaged in the learning of connection between actions and reward consequences^34^. The ACC may be involved in effort-based decision making^35^, i.e., how much effort should be exerted to obtain a reward in certain circumstances, and it also plays an important role in social interactions. It has been shown that lesions at the ACC could lead to a lower valuation of social information^36^ and a reduction in social behaviour^37^. Apart from the ACC, the precuneus is also involved in social cognition tasks^38^. In relation to WM, these three brain regions are central in filtering out irrelevant information^16^ and monitoring conflict and exerting executive efforts in demanding situations^39^. Brain dysfunctions in WM and affective processing across these regions have been reported in high-risk samples^40^ and patients with schizophrenia^41^,^42^. Brain activations were increased at the SFG and the supramarginal gyrus in the anticipation of potential monetary gain and at the MFG and IFG in the anticipation of monetary loss with WM training. The prefrontal cortex exerts top-down cognitive control and modulates the evaluation function of subcortical regions within the fronto-striatal rewarding system^43^. The enhanced brain activations associated with WM training during the anticipatory phase may reflect improved efficiency in modulating, coordinating and integrating goal-relevant information within the present task demand by activating the value presentation of cues. The presence of training effect at the dorsolateral prefrontal cortex and parietal regions is consistent with findings in a meta-analysis that evaluates brain activation associated with WM training^20^. The meta-analysis, however, indicated a decrease in activation in the prefrontal-parietal network when performing trained WM tasks. Brain activities when performing the transfer tasks, such as the untrained 3-back task^44^ and the emotion regulation task^45^, however, were elevated after training. Increased functional brain connectivity was observed between the MFG, the SFG and the ACC after WM training, and this increase was positively correlated with WM performance improvement^46^. We also found a positive correlation between activation at the SFG and *n*-back performance improvement. A previous study has found that WM training could reduce the discounting rate for future rewards in individuals with addiction^47^. A study using the AID task have shown that WM training could restore hedonic deficits towards social affective incentives in individuals with social anhedonia and their anticipatory sensitivity to affective rewards were normalized to the same level as individuals without hedonic processing deficits^21^. Thus the present study provides additional evidence for the neural mechanism underlying the improvement of hedonic processing ability and supports the efficacy of WM training in non-clinical samples.

Interestingly, after a direct comparison of training benefits across the anticipatory and consummatory phase of the AID and the MID tasks, we found a consistent pattern of increased brain activation in the anticipatory phase and decreased brain activation in the consummatory phase in both tasks. However, the involved brain regions differed between these two tasks. Behaviourally, consummatory pleasure capacity has been reported to be intact in individuals with social anhedonia and patients with schizophrenia^9^. WM is primarily involved in the anticipation of future rewards and helps to facilitate goal-directed behaviours^13^. The training effect observed in the AID and the MID tasks could be interpreted as an optimization of brain functions for information processing within WM. The improved maintenance of information about the rewarding outcomes of cues and actions could facilitate more adaptive responses. Behavioural studies did not identify any hedonic processing deficits toward monetary rewards in individuals with social anhedonia^22^ and the AID task has also been suggested to be more sensitive in detecting physical anhedonia^23^. The observed dissociable training effects between the AID and the MID tasks further support the domain-specific characteristic of hedonic processing.

Augmenting hedonic processing ability through simple, easily administered computerized WM training may provide a novel approach to alleviate anhedonia not only in high-risk individuals but also in patients with schizophrenia. Evidence of the neural restorative effect of cognitive remediation has been reported in patients with schizophrenia^48^. Brain activities have been reported to normalize to that of healthy controls at key regions responsible for WM^49^, and increased fractional anisotropy (FA) has been observed in the anterior part of the genu of the corpus callosum after 40 sessions of WM training in patients with schizophrenia^50^. Neural transfer effects have also been reported in restoring brain functions related to reality monitoring^51^ and emotion recognition^52^, indicating a potential for improving general functional outcomes with cognitive training in patients with schizophrenia. However, due to the small sample size and lack of a control group in this study, our results remain preliminary and require replication in future research.

The present study had several limitations. First, the sample size of the social anhedonia group was relatively small, which might limit the interpretability of the results. Secondly, the number of trials in each condition was relatively small to avoid fatigue for participants. However, there has been evidence for the distinctness of neural processing for monetary and affective rewards^23^. Thirdly, the control group was tested during the baseline assessment only, and the training effect observed in the social anhedonia group might be due to practice effect. Finally, only neural measures of hedonic processing were included, and the training effect on WM processing was only investigated with behavioural tasks. Thus the common neural mechanism of WM and hedonic processing was not thoroughly investigated in this study.

Notwithstanding these limitations, the present study adds significantly to the extant literature on the plasticity of hedonic processing. We demonstrate that WM training could enhance brain activations during reward anticipation in individuals with social anhedonia. These findings also suggest the potential of developing non-pharmacological interventions to alleviate anhedonia in patients with schizophrenia and depression.

## Methods

### Participants

Individuals with social anhedonia were recruited from a large sample consisting of 700 college students from a university based on scores on the Chapman Social Anhedonia Scale (CSAS)^53^,^54^. Individuals scoring above 1.96 *SD* of the same-sex norm mean^54^ (female: >18; male: >21) were invited to participate in the training programme. Individuals with a history of psychiatric disorders, brain injury, neurological disorders or substance abuse were excluded. Seventeen individuals with social anhedonia completed the pre-training assessment, but two of them did not complete the whole set of training due to personal reasons. The remaining 15 participants took part in 20 sessions of WM training, with each session lasting for approximately 30 minutes. They also took part in the pre- and post-training assessment of WM capacity and emotional experience. Two functional imaging versions of the MID and the AID tasks were incorporated in the pre- and post-training sessions to assess any potential improvement in hedonic processing ability.

**Figure 4:**
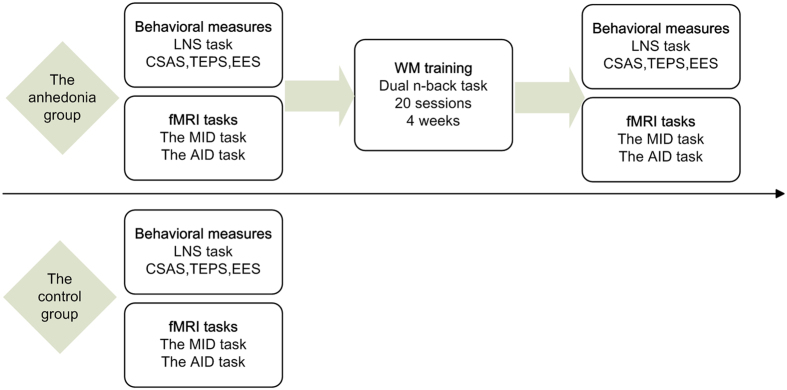
Schematic diagram of experimental procedure. LNS task: Letter Number Span task; CSAS: Chapman Social Anhedonia Scale; TEPS: Temporal Experience of Pleasure Scale; EES: Emotional Expressivity Scale; AID: Affective Incentive Delay; MID: Monetary Incentive Delay.

In addition, we included a group of individuals with low levels of social anhedonia as controls to detect any baseline brain dysfunctions and training-related improvement in the anhedonia group. These participants (*n* = 19) were individuals with a CSAS score no higher than 0.5 *SD* of the same-sex norm mean (female: <13; male: <11) and they only participated in the baseline assessment, which consisted of both behavioural and neuroimaging measures similar to individuals with social anhedonia. A schema of the experimental procedures is presented in Fig. 4. The two groups were matched in gender, age, education and WM capacity (see Supplementary Table S4). Individuals with social anhedonia reported higher level of anhedonia, less pleasure when anticipating future events, and rated themselves to be less emotionally expressive.

The study was approved by the Ethics Committee of the Institute of Psychology, the Chinese Academy of Sciences and was carried out in accordance with the approved guidelines. Written informed consents were obtained from all participants.

### Training arrangement

**Figure 5:**
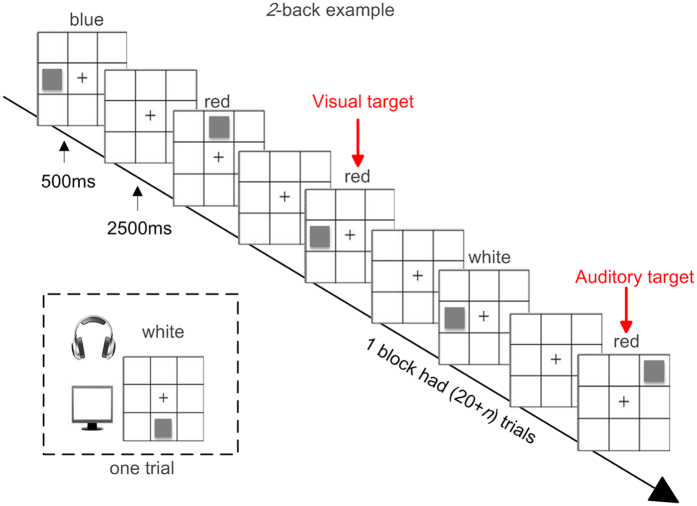
A *2*-back example of the dual *n*-back task.

The WM training task was a dual *n*-back task adapted from Jaeggi *et al*.^55^. Chinese characters with one syllable were used as the auditory stimulus rather than English letters (Fig. 5). Participants were asked to decide whether the location of a square and the character they heard were the same as the one *n*-back before. Each training session had 20 blocks, and each block had 20 + *n* trials, which included six auditory and six visual targets (four auditory or visual target trials separately and two trials of both an auditory target and a visual target), where *n* was the same for both modalities. If a target was correctly detected, a single tone was given, which constituted a positive feedback.

The training task was designed to continuously adjust its difficulty by changing the working memory load, which was represented by *n*. Each training session started at *n* = 1. If five or more targets were correctly detected in each modality, *n* would increase by one in the next block. Conversely, *n* would decrease by one if more than four mistakes in either modality were made. The *n* remained unchanged in other cases. Each training session lasted for 20 to 30 minutes, and participants were asked to come to the laboratory five days a week for four weeks.

### Behavioural assessment at pre-training and post-training

To assess the training effect on general WM capacity, the Letter-Number-Span Task (LNS)^56^,^57^ was administered to all participants. The total number of items successfully passed was used as the dependent variable.

All participants were also asked to complete two self-report scales that measure one’s emotional feelings and emotional expressivity. The Temporal Experience of Pleasure Scale (TEPS)^1^,^58^ was used to assess individual trait dispositions in both anticipatory and consummatory pleasure. Higher scores indicated better hedonic capacity. The Emotional Expressivity Scale (EES)^59^,^60^, consisting of two factors, “emotion expression” and “emotion suppression”, was used to assess emotional expressivity.

### Neuroimaging tasks

The participants were administered two incentive delay tasks, namely the MID and the AID tasks. These two tasks are designed to capture both anticipatory and consummatory pleasure processing but differ in terms of the type of stimulus (monetary vs. affective) used. The details of these two tasks have been described elsewhere^22^,^23^,^24^. A cue was first presented, which was followed by a delay period, and participants were instructed to press a specified key as quickly as possible when the target appeared. A feedback (win, loss, no loss or no win) was then given. Both tasks had two runs, with each run consisting of 30 trials, with 10 reward, punishment and neutral conditions respectively. The order of all trials in each run was pseudo-randomly arranged.

In the beginning of each trial, participants saw one of three cues (circle, triangle or square) for 250 ms at the centre of the computer screen. The cue period was followed by a delay period lasting for 2000 ms to 2500 ms, which was designed to capture the anticipatory phase. Then a target appeared and participants were asked to respond as quickly as possible with their right index finger by pressing a specified key. There was a feedback period of 1650 ms for the monetary incentives or 3000 ms for the affective incentives following the disappearance of the target, notifying the participants whether they had hit the target or not during that trial. In the MID task, monetary incentive was given as feedback, and the money they won would become part of the monetary compensation they received after the experiment. A triangular cue indicated a reward condition, in which if the participants successfully hit the target, the incentive that followed would show that they had won five points. A square cue indicated a punishment condition, in which if the participants did not hit the target, they would lose five points; if they hit the target successfully, they could avoid the loss. A circular cue meant a neutral condition, in which no monetary reward or punishment was given no matter whether the participant hit the target or not.

In the AID task, participants received affective pictures as feedback during the consummation period. Under the reward condition (triangular cue), participants were presented with a positive affective image if their reaction times (RT) were sufficiently short. Under the punishment condition (square cue), participants would see a negative affective image as feedback if they did not hit the target. Otherwise a neutral image was shown if they responded before the target disappeared. Under the neutral condition (circle cue), participants were shown a neutral image as feedback regardless of RTs. These images were selected from the International Affective Picture System (IAPS)^61^ and have been validated in terms of valence and arousal in a previous study^22^. The feedback period of the AID task lasted for 3000 ms to facilitate the emotion-eliciting effect of the affective images.

### Images acquisition and preprocessing

All Magnetic Resonance Imaging (MRI) scans were acquired using a 3-Tesla scanner system (MAGNETOM^®^ Verio Siemens). A high-resolution T1-weighted structural image was acquired with the following parameters: repetition time (TR) = 2,300 ms, echo time (TE) = 3 ms, field of view (FOV) = 256 mm, flip angle = 9°, image matrix = 256 × 256, and voxel dimensions = 1 mm × 1 mm × 1 mm. Functional images were acquired with echo planar imaging sequence, with the following parameters: TR = 2,000 ms, TE = 30 ms; FOV = 210 mm, slices = 32, flip angle = 90°, image matrix = 64 × 64, and voxel dimensions = 3.3 mm × 3.3 mm × 4 mm. Each run had 184 whole-brain volumes, such that each task had a total of 368 volumes. The sequence of the MID and AID tasks was counterbalanced across participants. A T2-weighted image was acquired to exclude participants with organic brain disease.

Pre-processing was performed using the Statistical Parametric Mapping software (SPM8, Wellcome Department of Imaging Neuroscience, Institute of Neurology and the National Hospital for Neurology and Neurosurgery; London, England). The data were first realigned with respect to the first volume to correct for head motion. Next, the Artifact detection Tool (ART) was used to generate a regressor to account for images with significant movement (scan to scan movement threshold 0.6 mm) or spike artifacts (absolute movement greater than 3 mm). Data from participants with more than 10% variation were excluded. Thus one participant in the anhedonia group was excluded in the subsequent imaging data analysis. Functional images were then corrected for slice timing and co-registered to the structural image of each participant, and then re-sampled into 3 mm × 3 mm × 3 mm slices. Finally, all normalized images were spatially smoothed with 8-mm full-width at half maximum Gaussian Kernel to remove low-frequency noise.

### Statistical analyses

#### Behavioural data

Statistical analyses were performed using SPSS (17.0 for Windows, SPSS Inc., Chicago, IL, USA). Training effects on the LNS task and self-report measures were analyzed with paired *t*-test (two tailed), while the differences between groups were analyzed with two sample *t*-tests (two tailed).

#### Imaging data

In the first-level analysis, a haemodynamic response function with nine conditions (reward cue, punishment cue, neutral cue, reward cue hit, reward cue miss, punishment cue hit, punishment cue miss, neutral cue hit, neutral cue miss) and seven head movement parameters (six re-alignment parameters and one ART regressor) as covariates of no interest were modeled separately for each task and each MRI session. In the second-level analysis, the contrast “reward cue > neutral cue” and “punishment cue > neutral cue” were established to identify brain activation during the anticipation of the positive and negative incentives respectively. The contrasts “reward cue hit > neutral cue hit” and “punishment cue miss > neutral cue miss” were established to identify brain activation during the consummation of reward and punishment respectively. To estimate activation changes across time, these contrasts were then compared before and after training using paired *t*-tests on SPM8, while the difference between individuals with social anhedonia and controls were evaluated using two-sample *t*-tests on SPM8. For the whole brain group-level analysis, the significant threshold of each contrast was set as *p* < 0.001 (uncorrected), with a spatial extent threshold of 10 contiguous voxels.

## Additional Information

**How to cite this article**: Li, X. *et al*. The neural transfer effect of working memory training to enhance hedonic processing in individuals with social anhedonia. *Sci. Rep.* **6**, 35481; doi: 10.1038/srep35481 (2016).

## Supplementary Material

Supplementary Information

## Figures and Tables

**Table 1:** Effects of working memory training on brain activations in the anticipation phase of the AID and the MID task in individuals with social anhedonia (*n* = 14).

Contrast	Numbers of sig. clusters	Cluster size (voxels)	x,y,z (MNI Coordinate)	Peak *t*	Brain regions	
**AID task: anticipation phase**						
reward cue > neutral cue						
pre > post	0					
post > pre	3	29	12;24;15	6.41	Anterior Cingulate Cortex	
			0;30;15	4.26		
		11	−21;18;9	6.27	Dorsal Striatum	
		19	−33;−72;39	4.96	Precuneus	
punishment cue > neutral cue						
pre > post	0					
post > pre	0					
**MID task: anticipation phase**						
reward cue > neutral cue						
pre > post	0					
post > pre	2	40	9;18;66	3.82	Superior Frontal Gyrus	
			−3;21;63	3.68		
			−6;15;69	3.48		
		19	63;−39;39	3.61	Supramarginal Gyrus	
punishment cue > neutral cue						
pre > post	0					
post > pre	2	10	30;42;15	5.22	Middle Frontal Gyrus	
		14	−42;15;−15	4.75	Inferior Frontal Gyrus	

pre > post: decreased brain activations related with working memory training; post > pre: increased brain activations related with working memory training.

## References

[b1] Gard D. E., Kring A. M., Gard M. G., Horan W. P. & Green M. F. Anhedonia in schizophrenia: distinctions between anticipatory and consummatory pleasure. Schizophr Res. 93, 253 (2007). 17490858 10.1016/j.schres.2007.03.008 PMC1986826

[b2] Keedwell P. A., Andrew C., Williams S. C. R., Brammer M. J. & Phillips M. L. The neural correlates of anhedonia in major depressive disorder. Biol Psychiatry 58, 843–853 (2005). 16043128 10.1016/j.biopsych.2005.05.019

[b3] Cuthbert B. N. The RDoC framework: facilitating transition from ICD/DSM to dimensional approaches that integrate neuroscience and psychopathology. World Psychiatry 13, 28–35 (2014). 24497240 10.1002/wps.20087 PMC3918011

[b4] Blanchard J. J., Mueser K. T. & Bellack A. S. Anhedonia, positive and negative affect, and social functioning in schizophrenia. Schizophr Bull. 24, 413–424 (1998). 9718633 10.1093/oxfordjournals.schbul.a033336

[b5] Ritsner M. S., Arbitman M. & Lisker A. Anhedonia is an important factor of health-related quality-of-life deficit in schizophrenia and schizoaffective disorder. J Nerv Ment Dis. 199, 845–853 (2011). 22048136 10.1097/NMD.0b013e3182349ce6

[b6] Docherty A. R. & Sponheim S. R. Anhedonia as a phenotype for the Val¹ Met COMT polymorphism in relatives of patients with schizophrenia. J Abnorm Psychol. 117, 788–798 (2008). 19025226 10.1037/a0013745 PMC2936689

[b7] Wang Y. . Individuals with psychometric schizotypy show similar social but not physical anhedonia to patients with schizophrenia. Psychiatry Res. 216, 161–167 (2014). 24589449 10.1016/j.psychres.2014.02.017

[b8] Kendler K. S. & Neale M. C. Endophenotype: a conceptual analysis. Mol Psychiatry 15, 789–797 (2010). 20142819 10.1038/mp.2010.8 PMC2909487

[b9] Li Z. . Experiential pleasure deficits in different stages of schizophrenia. Schizophr Res. 166, 98–103 (2015). 26072322 10.1016/j.schres.2015.05.041

[b10] Gold J. M., Waltz J. A., Prentice K. J., Morris S. E. & Heerey E. A. Reward processing in schizophrenia: a deficit in the representation of value. Schizophr Bull. 34, 835–847 (2008). 18591195 10.1093/schbul/sbn068 PMC2518641

[b11] Gard D. E. . Evidence for an emotion maintenance deficit in schizophrenia. Psychiatry Res. 187, 24–29 (2011). 21237516 10.1016/j.psychres.2010.12.018 PMC3070787

[b12] Ursu S. . Prefrontal cortical deficits and impaired cognition-emotion interactions in schizophrenia. Am J Psychiatry 168, 276–285 (2011). 21205806 10.1176/appi.ajp.2010.09081215 PMC4019338

[b13] Kring A. M. & Caponigro J. M. Emotion in schizophrenia: where feeling meets thinking. Curr Dir Psychol Sci. 19, 255–259 (2010). 22557707 10.1177/0963721410377599 PMC3340922

[b14] Baddeley A., Banse R., Huang Y.-M. & Page M. Working memory and emotion: Detecting the hedonic detector. J Cogn Psychol. 24, 6–16 (2012).

[b15] Barch D. M. & Dowd E. C. Goal representations and motivational drive in schizophrenia: the role of prefrontal–striatal interactions. Schizophr Bull. 36, 919–934 (2010). 20566491 10.1093/schbul/sbq068 PMC2930335

[b16] McNab F. & Klingberg T. Prefrontal cortex and basal ganglia control access to working memory. Nat Neurosci. 11, 103–107 (2008). 18066057 10.1038/nn2024

[b17] Blanchard J. J., Collins L. M., Aghevli M., Leung W. W. & Cohen A. S. Social anhedonia and schizotypy in a community sample: the Maryland longitudinal study of schizotypy. Schizophr Bull. 37, 587–602 (2011). 19850669 10.1093/schbul/sbp107 PMC3080671

[b18] Kwapil T. R. Social anhedonia as a predictor of the development of schizophrenia-spectrum disorders. J Abnorm Psychol. 107, 558–565 (1998 9830243 10.1037//0021-843x.107.4.558

[b19] Kurtz M. M. Cognitive remediation for schizophrenia: current status, biological correlates and predictors of response. Expert Rev of Neurother. 12, 813–821 (2012). 22853789 10.1586/ern.12.71 PMC11744976

[b20] Li X. . The neuroplastic effect of working memory training in healthy volunteers and patients with schizophrenia: Implications for cognitive rehabilitation. Neuropsychologia 75, 149–162 (2015). 26032579 10.1016/j.neuropsychologia.2015.05.029

[b21] Li X. . The effects of working memory training on enhancing hedonic processing to affective rewards in individuals with high social anhedonia. Psychiatry Res. 10.1016/j.psychres.2016.09.006 (2016). 27639163

[b22] Xie W.-z. . Domain-specific hedonic deficits towards social affective but not monetary incentives in social anhedonia. Sci Rep. 4 (2014). 10.1038/srep04056 PMC4070222 24514898

[b23] Chan R. C. . Distinct processing of social and monetary rewards in late adolescents with trait anhedonia. Neuropsychology 30, 274–280 (2016). 26280299 10.1037/neu0000233

[b24] Knutson B., Adams C. M., Fong G. W. & Hommer D. Anticipation of increasing monetary reward selectively recruits nucleus accumbens. J Neurosci. 21, RC159 (2001). 11459880 10.1523/JNEUROSCI.21-16-j0002.2001 PMC6763187

[b25] Blakemore S.-J. The social brain in adolescence. Nat Rev Neurosci. 9, 267–277 (2008). 18354399 10.1038/nrn2353

[b26] Wang Y. . Dimensional schizotypy and social cognition: an fMRI imaging study. Front Behav Neurosci. 9, 133 (2015). 26074796 10.3389/fnbeh.2015.00133 PMC4444828

[b27] Brüne M. . An fMRI study of theory of mind in schizophrenic patients with “passivity” symptoms. Neuropsychologia 46, 1992–2001 (2008). 18329671 10.1016/j.neuropsychologia.2008.01.023

[b28] Camara E., Rodriguez-Fornells A. & Munte T. F. Functional connectivity of reward processing in the brain. Front Hum Neurosci. 2, 19 (2008). 19242558 10.3389/neuro.09.019.2008 PMC2647336

[b29] Sescousse G., Caldú X., Segura B. & Dreher J.-C. Processing of primary and secondary rewards: a quantitative meta-analysis and review of human functional neuroimaging studies. Neurosci Biobehav Rev. 37, 681–696 (2013). 23415703 10.1016/j.neubiorev.2013.02.002

[b30] Arias-Carrion O., Stamelou M., Murillo-Rodriguez E., Menendez-Gonzalez M. & Poppel E. Dopaminergic reward system: a short integrative review. Int Arch Med. 3, 24 (2010). 20925949 10.1186/1755-7682-3-24 PMC2958859

[b31] Juckel G. . Dysfunction of ventral striatal reward prediction in schizophrenia. Neuroimage 29, 409–416 (2006). 16139525 10.1016/j.neuroimage.2005.07.051

[b32] Zhang W.-N., Chang S.-H., Guo L.-Y., Zhang K.-L. & Wang J. The neural correlates of reward-related processing in major depressive disorder: A meta-analysis of functional magnetic resonance imaging studies. J Affec Disord. 151, 531–539 (2013). 10.1016/j.jad.2013.06.039 23856280

[b33] Rademacher L. . Dissociation of neural networks for anticipation and consumption of monetary and social rewards. Neuroimage 49, 3276–3285 (2010). 19913621 10.1016/j.neuroimage.2009.10.089

[b34] O’Doherty J. . Dissociable roles of ventral and dorsal striatum in instrumental conditioning. Science 304, 452–454 (2004). 15087550 10.1126/science.1094285

[b35] Salamone J. D., Correa M., Farrar A. & Mingote S. M. Effort-related functions of nucleus accumbens dopamine and associated forebrain circuits. Psychopharmacology (Berl) 191, 461–482 (2007). 17225164 10.1007/s00213-006-0668-9

[b36] Rudebeck P., Buckley M., Walton M. & Rushworth M. A role for the macaque anterior cingulate gyrus in social valuation. Science 313, 1310–1312 (2006). 16946075 10.1126/science.1128197

[b37] Hadland K. A., Rushworth M. F., Gaffan D. & Passingham R. E. The effect of cingulate lesions on social behaviour and emotion. Neuropsychologia 41, 919–931 (2003). 12667528 10.1016/s0028-3932(02)00325-1

[b38] Cavanna A. E. & Trimble M. R. The precuneus: a review of its functional anatomy and behavioural correlates. Brain 129, 564–583 (2006). 16399806 10.1093/brain/awl004

[b39] Nee D. E. . A meta-analysis of executive components of working memory. Cereb Cortex 23, 264–282 (2013). 22314046 10.1093/cercor/bhs007 PMC3584956

[b40] Dutt A. . Exploring neural dysfunction in ‘clinical high risk’ for psychosis: a quantitative review of fMRI studies. J Psychiatr Res. 61, 122–134 (2015). 25479766 10.1016/j.jpsychires.2014.08.018

[b41] Minzenberg M. J. & Carter C. S. Developing treatments for impaired cognition in schizophrenia. Trends Cogn Sci. 16, 35–42 (2012). 22178120 10.1016/j.tics.2011.11.017

[b42] Taylor S. F. . Meta-analysis of functional neuroimaging studies of emotion perception and experience in schizophrenia. Biol Psychiatry 71, 136–145 (2012). 21993193 10.1016/j.biopsych.2011.09.007 PMC3237865

[b43] Richards J. M., Plate R. C. & Ernst M. A systematic review of fMRI reward paradigms used in studies of adolescents vs. adults: the impact of task design and implications for understanding neurodevelopment. Neurosci Biobehav Rev. 37, 976–991 (2013). 23518270 10.1016/j.neubiorev.2013.03.004 PMC3809756

[b44] Dahlin E., Neely A. S., Larsson A., Bäckman L. & Nyberg L. Transfer of learning after updating training mediated by the striatum. Science 320, 1510–1512 (2008). 18556560 10.1126/science.1155466

[b45] Schweizer S., Grahn J., Hampshire A., Mobbs D. & Dalgleish T. Training the emotional brain: Improving affective control through emotional working memory training. J Neurosci. 33, 5301–5311 (2013). 23516294 10.1523/JNEUROSCI.2593-12.2013 PMC6704999

[b46] Jolles D. D., van Buchem M. A., Crone E. A. & Rombouts S. A. Functional brain connectivity at rest changes after working memory training. Hum Brain Mapp. 34, 396–406 (2013). 22076823 10.1002/hbm.21444 PMC6870317

[b47] Bickel W. K., Yi R., Landes R. D., Hill P. F. & Baxter C. Remember the future: Working memory training decreases delay discounting among stimulant addicts. Biol Psychiatry 69, 260–265 (2011). 20965498 10.1016/j.biopsych.2010.08.017 PMC3015021

[b48] Ramsay I. S. & MacDonald A. W. Brain correlates of cognitive remediation in schizophrenia: Activation likelihood analysis shows preliminary evidence of neural target engagement. Schizophr Bull. 41, 1276–1284 (2015). 25800249 10.1093/schbul/sbv025 PMC4601705

[b49] Bor J. . How can cognitive remediation therapy modulate brain activations in schizophrenia? An fMRI study. Psychiatry Res. 192, 160–166 (2011). 21543191 10.1016/j.pscychresns.2010.12.004

[b50] Penades R. . Brain effects of cognitive remediation therapy in schizophrenia: a structural and functional neuroimaging study. Biol Psychiatry 73, 1015–1023 (2013). 23452665 10.1016/j.biopsych.2013.01.017

[b51] Subramaniam K. . Computerized cognitive training restores neural activity within the reality monitoring network in schizophrenia. Neuron 73, 842–853 (2012). 22365555 10.1016/j.neuron.2011.12.024 PMC3295613

[b52] Hooker C. I. . The influence of combined cognitive plus social-cognitive training on amygdala response during face emotion recognition in schizophrenia. Psychiatry Res. 213, 99–107 (2013). 23746615 10.1016/j.pscychresns.2013.04.001 PMC6999046

[b53] Eckblad M., Chapman L., Chapman J. & Mishlove M. The revised social anhedonia scale. Unpublished test (1982).

[b54] Chan R. C. . A study of trait anhedonia in non-clinical Chinese samples: Evidence from the Chapman Scales for Physical and Social Anhedonia. PloS one 7, e34275 (2012). 22529910 10.1371/journal.pone.0034275 PMC3328477

[b55] Jaeggi S. M., Buschkuehl M., Jonides J. & Perrig W. J. Improving fluid intelligence with training on working memory. Proc Natl Acad Sci. 105, 6829–6833 (2008). 18443283 10.1073/pnas.0801268105 PMC2383929

[b56] Gold J. M., Carpenter C., Randolph C., Goldberg T. E. & Weinberger D. R. Auditory working memory and Wisconsin Card Sorting Test performance in schizophrenia. Arch Gen Psychiatry 54, 159–165 (1997). 9040284 10.1001/archpsyc.1997.01830140071013

[b57] Chan R. C. . The development of a Chinese equivalence version of letter-number span test. Clin Neuropsychol. 22, 112–121 (2008). 18247221 10.1080/13825580601025957

[b58] Chan R. C. . The Temporal Experience of Pleasure Scale (TEPS): exploration and confirmation of factor structure in a healthy Chinese sample. PloS one 7, e35352 (2012). 22530007 10.1371/journal.pone.0035352 PMC3329425

[b59] Kring A. M., Smith D. A. & Neale J. M. Individual differences in dispositional expressiveness: development and validation of the Emotional Expressivity Scale. J Pers Soc Psychol. 66, 934–949 (1994). 8014836 10.1037//0022-3514.66.5.934

[b60] Chan R. C. . A 2-stage factor analysis of the Emotional Expressivity Scale in the Chinese context. Psychologia 53, 44–50 (2010).

[b61] Lang P. J., Bradley M. M. & Cuthbert B. N. International Affective Picture System (IAPS): Technical Manual and Affective Ratings. (The Center for Research in Psychophysiology, University of Florida, 1997).

