# Supplementary material for: The neural transfer effect of working memory training to enhance hedonic processing in individuals with social anhedonia
Source: Sci Rep. 2016 Oct 18;6:35481. doi: 10.1038/srep35481 (PMC5067564; doi:10.1038/srep35481)
Supplement: Supplementary Information [file srep35481-s1.pdf]

The neural transfer effect of working memory training to enhance hedonic processing in individuals with social anhedonia

Xu Li[1,2,3],Zhi Li[1,2], Ke Li[4], Ya-wei Zeng[4], Hai-song Shi[1,2,5], Wen-lan Xie[1,2], Zhuo-ya Yang[1,2], ,Simon S. Y. Lui[1,2,6], Eric F. C. Cheung[6], Ada W. S. Leung[7], Raymond C. K. Chan[1,2*]

[1] Neuropsychology and Applied Cognitive Neuroscience Laboratory, CAS Key Laboratory of Mental Health, Institute of Psychology, Beijing, 100101, China

[2] University of Chinese Academy of Sciences, Beijing, 100048, China

[3] Key Laboratory of Adolescent Cyberpsychology and Behavior(CCNU), Ministry of Education, School of Psychology, Central China Normal University, Wuhan, 430079, China

[4] MRI Center, Hospital 306, 100101, Beijing, China

[5] North China Electric Power University, Beijing, 102206, China

[6] Castle Peak Hospital, Hong Kong Special Administration Region, China

[7] Department of Occupational Therapy & Institute of Neuroscience and Mental Health, University of Alberta, T6G 2G4, Canada

Supplementary Materials

Supplementary Table S1 Behavioral performances at the pre- and post-training sessions within the anhedonia group (*n* = 15)

	Pre-training session		Post-training session		*t*	*p*
	Mean	*SD*	*Mean*	*SD*		
LNS total	19.53	3.72	22.67	3.24	3.662	0.003
CSAS	22.67	3.27	18.8	6.06	2.303	0.037
TEPS	70.53	13.06	70.8	12.75	0.177	0.862
Anticipatory pleasure	30.07	7.96	29.6	7.6	0.392	0.701
Consummatory pleasure	40.47	5.66	41.2	5.82	1.244	0.234
EES	47.2	12.8	50.47	11.69	1.214	0.245
Expression	14.4	5.84	14.8	4.93	0.313	0.759
Suppression	32.8	8.43	35.67	7.53	1.705	0.11

LNS: Letter Number Span task; CSAS: Chapman Social Anhedonia Scale; TEPS: Temporal Experience of Pleasure Scale; EES: Emotional expressivity Scale. For the suppression subscale, lower score represents severe suppression from expressivity.

Supplementary Table S2 Comparisons of brain activation differences in the anticipation and consummation phases of the AID and the MID tasks between the anhedonia (n = 14) and controls (n = 19)

conditions	contrast	numbers of sig. clusters	cluster size	x,y,z (MNI Coordinate)	peak t	brain regions
**AID task: anticipation phase**						
reward cue>neutral cue						
	controls>SA	0				
	SA>controls	2	12	42 -63 24	4.40	Middle Temporal Gyrus
punishment cue>neutral cue			12	15 -60 24	3.79	Precuneus
	controls>SA	0				
	SA>controls	0				
**AID task: consummation phase**						
reward cue hit > neutral cue hit						
	controls>SA	0				
	SA>controls	5	86	-57;12;-6	4.94	Superior Temporal Gyrus
				-45;6;-6	4.87	Insula
			46	57;-42;36	4.41	Inferior Parietal Lobule
				60;-45;48	4.01	
			12	54;-21;-9	4.11	Superior Temporal Gyrus
			11	33;51;6	4.02	Middle Frontal Gyrus
			12	54;-57;39	3.66	Inferior Parietal Lobule
punishment cue miss > neutral cue miss						
	controls>SA	0				
	SA>controls	0				
**MID task: anticipation phase**						
reward cue>neutral cue						
	controls>SA	3	100	-27;-45;-6	5.41	Parahippocampus
				-33;-36;6	3.65	
			82	24;-33;-12	4.60	Parahippocampus
				30;-39;-6	3.78	
				21;-45;-15	3.59	
			68	-6;-30;-6	4.14	Thalamus
				3;-30;-6	3.92	
				9;-24;-6	3.90	
	SA>controls	0				
punishment cue>neutral cue						
	controls>SA	1	13	9;-24;-9	3.68	substantia nigra
	SA>controls	1	14	-54;-45;51	4.09	Inferior Parietal Lobule
**MID task: consummation phase**						
reward cue hit > neutral cue hit						
	controls>SA	0				
	SA>controls	0				
punishment cue miss > neutral cue miss						

controls>SA	0				
SA>controls	8	24	39;45;-6	4.56	Inferior Frontal gyrus
		63	21;45;18	4.53	Medial Frontal Gyrus
			27;42;12	4.30	
			21;54;12	3.93	
		29	0;-78;-15	4.39	Declive
			-6;-84;-21	3.75	
		35	6;-3;18	4.29	Thalamus
			0;3;15	4.23	
		15	57;21;21	4.20	Middle Frontal Gyrus
		44	-48;-72;-6	4.05	Fusiform Gyrus
			-39;-63;-15	3.69	
			-45;-63;-6	3.50	
		36	-57;-48;21	3.94	Supramarginal Gyrus
			-57;-48;30	3.86	
		21	-24;-81;-24	3.79	Uvula
			-12;-84;-21	3.52	

SA: social anhedonia.

Control>SA: greater brain activations in the controls than individuals with social anhedonia.

SA>controls: greater brain activations in individuals with social anhedonia compared with controls.

Supplementary Table S3 effects of working memory training on brain activations in the consummation phase of the AID and the MID task in individuals with social anhedonia (*n* = 14).

	contrast	numbers of sig. clusters	cluster size	x,y,z (MNI Coordinate)	peak t	brain regions
**AID task: consummation phase**						
reward cue hit > neutral cue hit						
	pre>post	16	98	24;-39;66	8.03	Superior Parietal Lobule
				18;-27;63	6.73	
				15;-15;69	5.05	
			763	-27;-15;24	7.20	Insula
				-15;-21;18	7.16	Caudate Tail
				0;15;12	7.02	Caudate Body
			68	-6;-90;36	6.53	Cuneus
				-12;-90;30	6.48	
			17	27;60;18	6.49	Superior Frontal Gyrus
			33	30;-54;3	6.09	Parahippocampus
			24	-21;-27;60	5.73	Postcentral Gyrus
				-18;-30;72	4.29	
			21	12;-81;42	5.64	Cuneus
				21;-84;36	4.22	
				18;-90;30	4.19	
			192	-12;-21;45	5.43	Cingulate
				-12;-6;45	5.32	
				3;-24;42	5.19	
			31	-33;-69;-30	5.37	Tuber
				-42;-60;-33	4.61	
			11	39;-54;-33	5.31	Culmen
			11	-9;9;42	5.08	Cingulate Cortex
			29	51;-21;9	5.03	
			11	27;33;39	4.97	Middle Frontal Gyrus
			45	-63;-36;12	4.93	Superior Temporal Gyrus
				-57;-39;18	4.54	
				-69;-42;21	4.52	
			11	30;-15;45	4.87	Precentral Gyrus
				24;-6;42	4.59	
			10	48;-12;42	4.76	Precentral Gyrus
				42;-12;33	4.19	
	post>pre	0				
punishment cue miss > neutral cue miss						
	pre>post	0				
	post>pre	0				
**MID task: consummation phase**						
reward cue hit > neutral cue hit						

	pre>post	1	21	-54;-66;-12	6.37	Fusiform Gyrus
				-48;-72;-12	5.67	
	post>pre	0				
punishment cue miss > neutral cue miss						
	pre>post	2	11	-39;-45;-9	6.13	Parahippocampus
			14	36;9;30	4.49	Precentral Gyrus
				39;18;33	3.93	
	post>pre	0				

pre>post: decreased brain activations related with working memory training;

Post>pre: increased brain activations related with working memory training;

Supplementary Table S4 Demographic information and other characteristics of the social anhedonia and controls

	Social Anhedonia	Controls	*t/χ2*	*p*
	Mean(SD)	*Mean(SD)*		
Gender (total/male)	15/9	19/11	0.015	0.901
Age	20.13(1.25)	19.63(2.14)	0.855	0.399
Education	13.8(0.94)	12.95(1.68)	1.869	0.072
LNS total	19.53(3.72)	17.47(2.5)	1.927	0.063
CSAS	22.67(3.27)	6.23(2.20)	15.348	0.000
TEPS	70.53(13.06)	83.31(11.46)	-2.730	0.011
Anticipatory pleasure	30.07(7.96)	36.23(5.8)	-2.308	0.029
Consummatory pleasure	40.47(5.66)	43.31(7.09)	-1.180	0.249
EES	47.2(12.8)	63.31(10.14)	-3.649	0.001
Expression	14.4(5.84)	16.92(3.86)	-1.325	0.197
Suppression	32.8(8.43)	46.38(7.95)	-4.365	0.000

LNS: Letter Number Span task; CSAS: Chapman Social Anhedonia Scale; TEPS: Temporal Experience of Pleasure Scale; EES: Emotional expressivity Scale. For the suppression subscale, lower score represents severe suppression from expressivity.
